# Supplementary material for: A Humanized Yeast Phenomic Model of Deoxycytidine Kinase to Predict Genetic Buffering of Nucleoside Analog Cytotoxicity
Source: Genes (Basel). 2019 Sep 30;10(10):770. doi: 10.3390/genes10100770 (PMC6826991; doi:10.3390/genes10100770)
Supplement: Supplementary file 1 [file genes-10-00770-s001.zip › Additional_Files/Additional_File7_GOTermHeatmaps.zip!/Additional_File7_GOTermHeatmaps/regulation_of_DNA_replication.pdf]

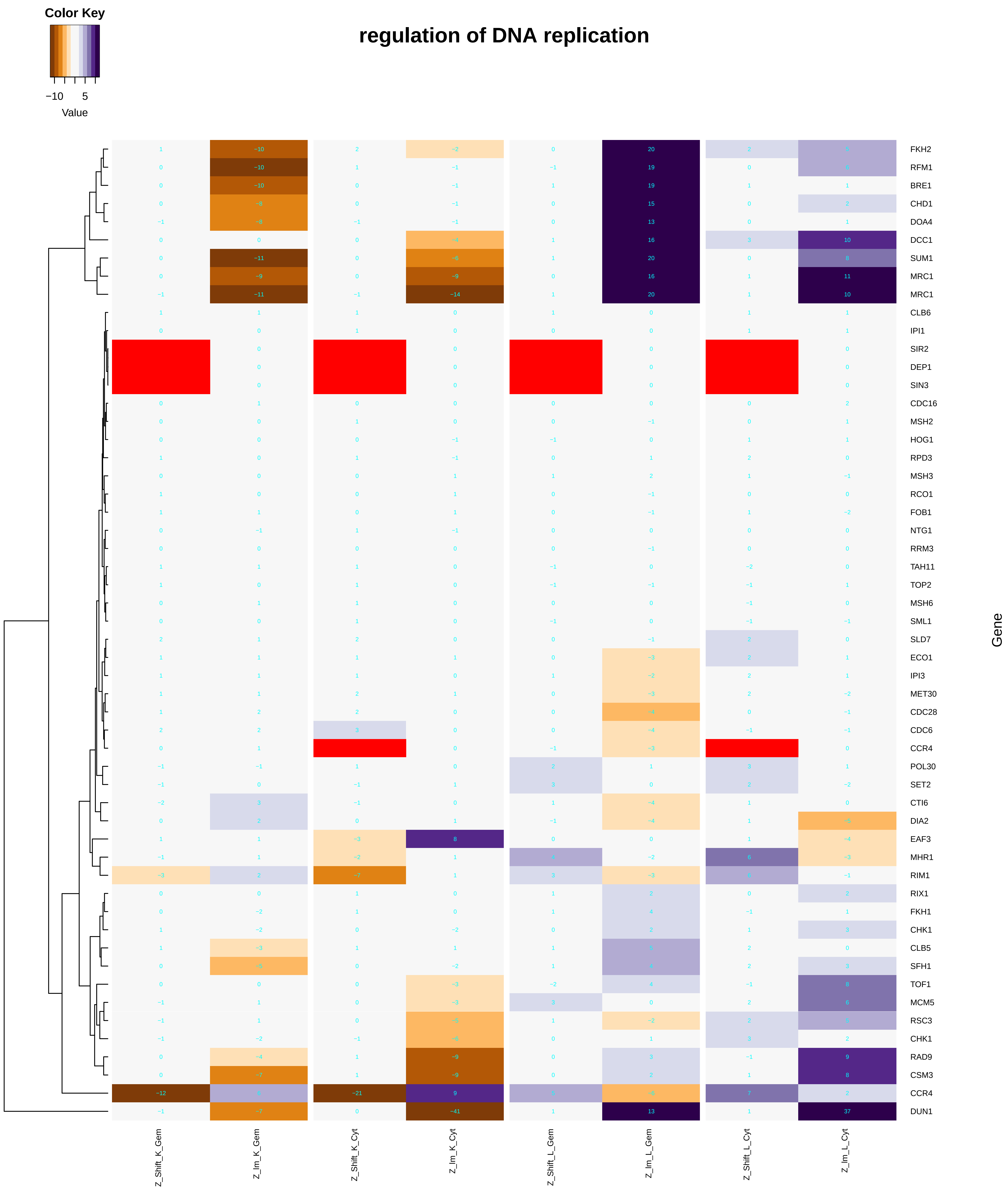
Color Key
−10 5
Value
regulation of DNA replication
FKH2
RFM1
BRE1
CHD1
DOA4
DCC1
SUM1
MRC1
MRC1
CLB6
IPI1
SIR2
DEP1
SIN3
CDC16
MSH2
HOG1
RPD3
MSH3
RCO1
FOB1
NTG1
RRM3
TAH11
TOP2
MSH6
SML1
SLD7
ECO1
IPI3
MET30
CDC28
CDC6
CCR4
POL30
SET2
CTI6
DIA2
EAF3
MHR1
RIM1
RIX1
FKH1
CHK1
CLB5
SFH1
TOF1
MCM5
RSC3
CHK1
RAD9
CSM3
CCR4
DUN1
Gene
Z_Shift_K_Gem
Z_Im_K_Gem
Z_Shift_K_Cyt
Z_Im_K_Cyt
Z_Shift_L_Gem
Z_Im_L_Gem
Z_Shift_L_Cyt
Z_Im_L_Cyt

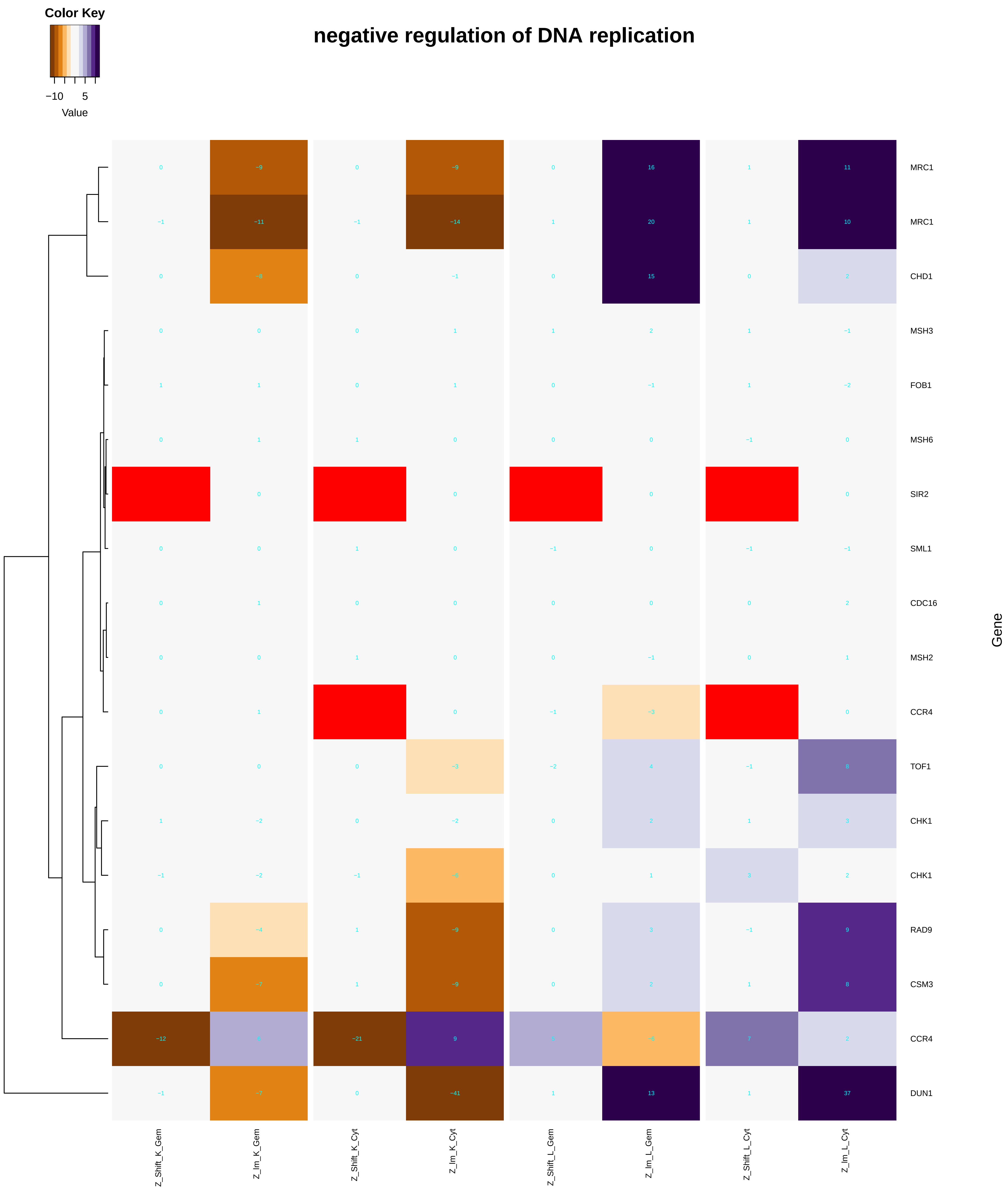
Color Key
−10
5
Value
negative regulation of DNA replication
MRC1
MRC1
CHD1
MSH3
FOB1
MSH6
SIR2
SML1
CDC16
MSH2
CCR4
TOF1
CHK1
CHK1
RAD9
CSM3
CCR4
DUN1
Gene
Z_Shift_K_Gem
Z_Im_K_Gem
Z_Shift_K_Cyt
Z_Im_K_Cyt
Z_Shift_L_Gem
Z_Im_L_Gem
Z_Shift_L_Cyt
Z_Im_L_Cyt

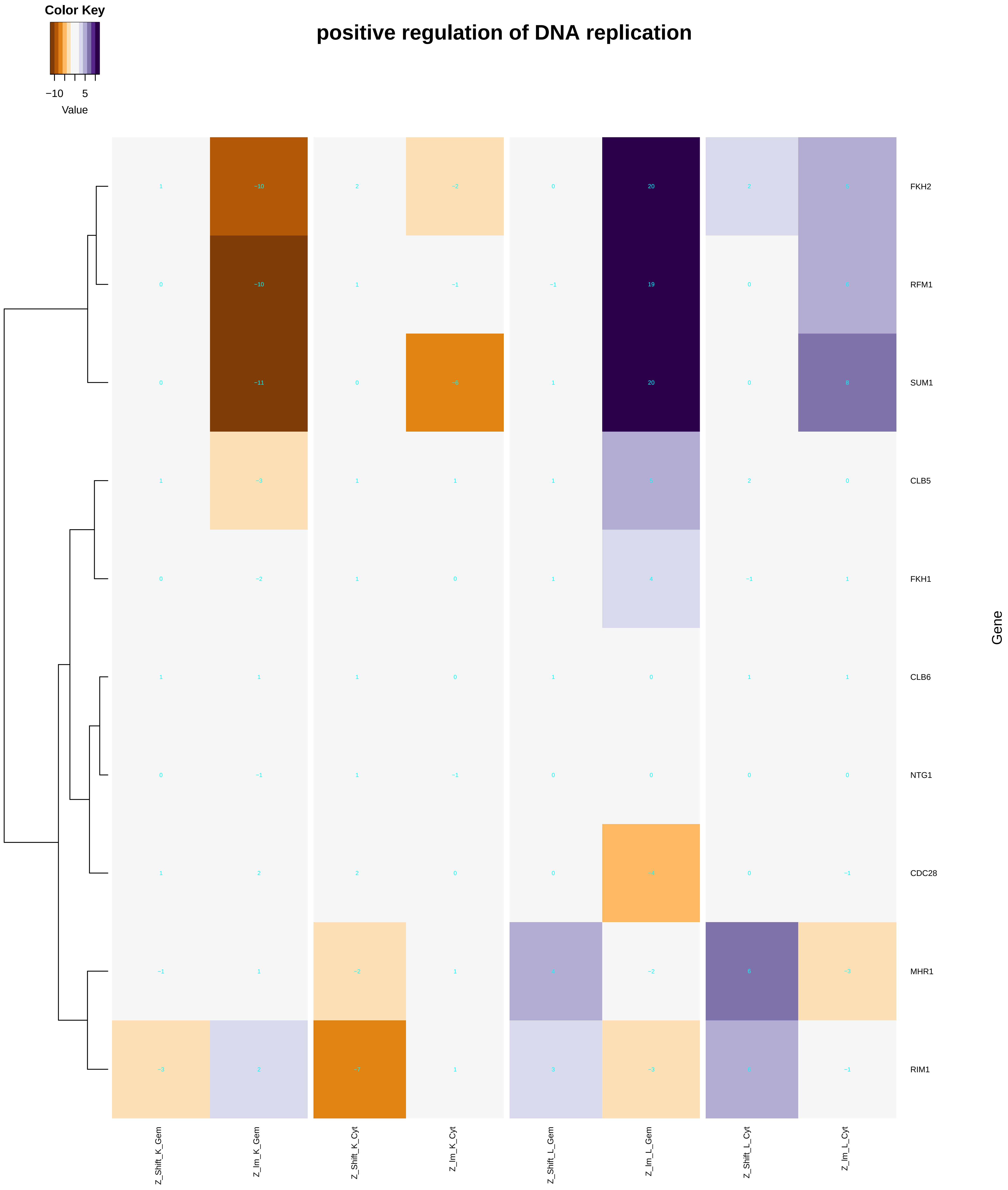
positive regulation of DNA replication
Color Key
−10
5
Value
FKH2
RFM1
SUM1
CLB5
FKH1
CLB6
NTG1
CDC28
MHR1
RIM1
Gene
Z_Shift_K_Gem
Z_Im_K_Gem
Z_Shift_K_Cyt
Z_Im_K_Cyt
Z_Shift_L_Gem
Z_Im_L_Gem
Z_Shift_L_Cyt
Z_Im_L_Cyt

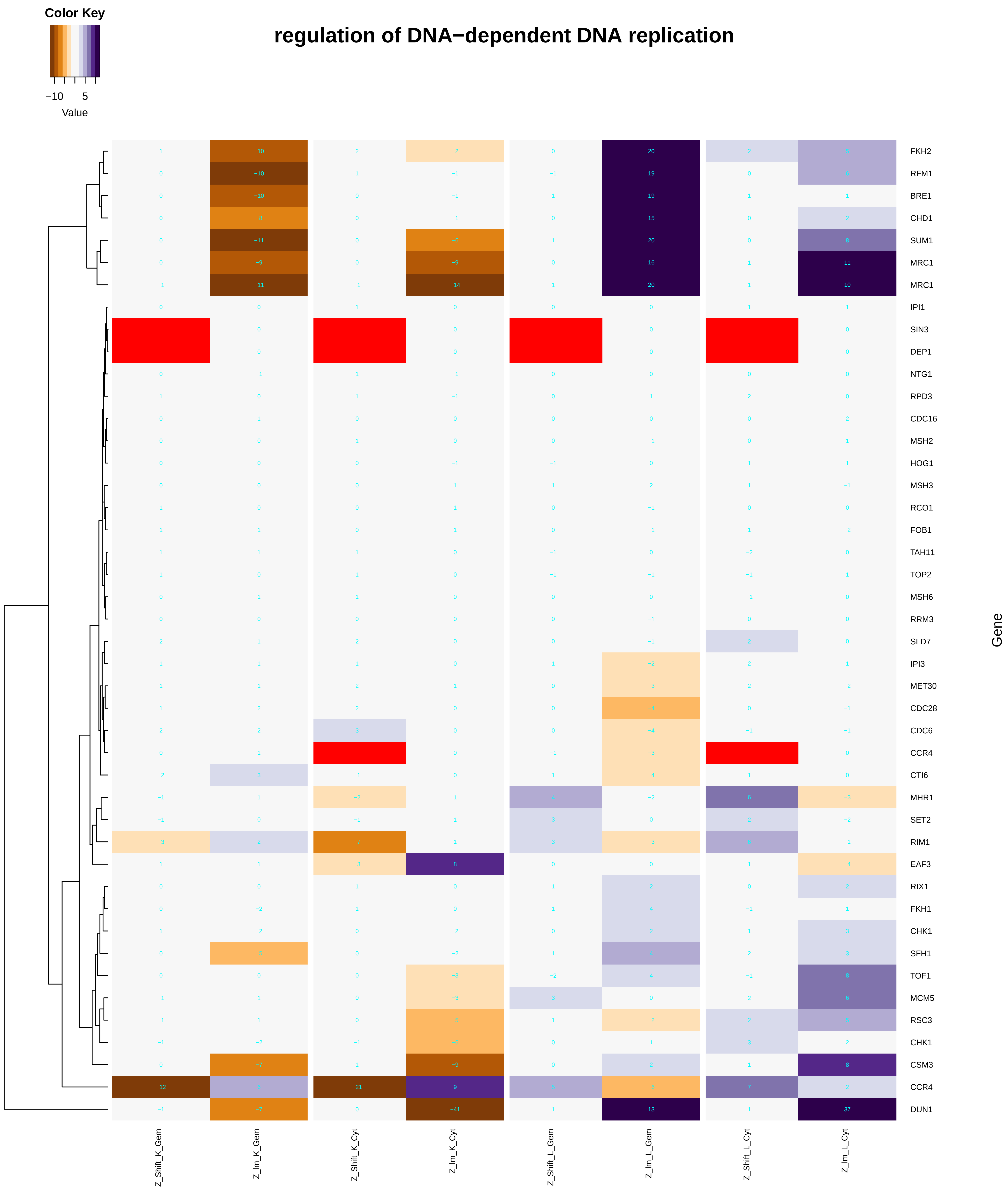
Color Key
−10 5
Value
regulation of DNA−dependent DNA replication
Gene
FKH2
RFM1
BRE1
CHD1
SUM1
MRC1
MRC1
IPI1
SIN3
DEP1
NTG1
RPD3
CDC16
MSH2
HOG1
MSH3
RCO1
FOB1
TAH11
TOP2
MSH6
RRM3
SLD7
IPI3
MET30
CDC28
CDC6
CCR4
CTI6
MHR1
SET2
RIM1
EAF3
RIX1
FKH1
CHK1
SFH1
TOF1
MCM5
RSC3
CHK1
CSM3
CCR4
DUN1
Z_Shift_K_Gem
Z_Im_K_Gem
Z_Shift_K_Cyt
Z_Im_K_Cyt
Z_Shift_L_Gem
Z_Im_L_Gem
Z_Shift_L_Cyt
Z_Im_L_Cyt

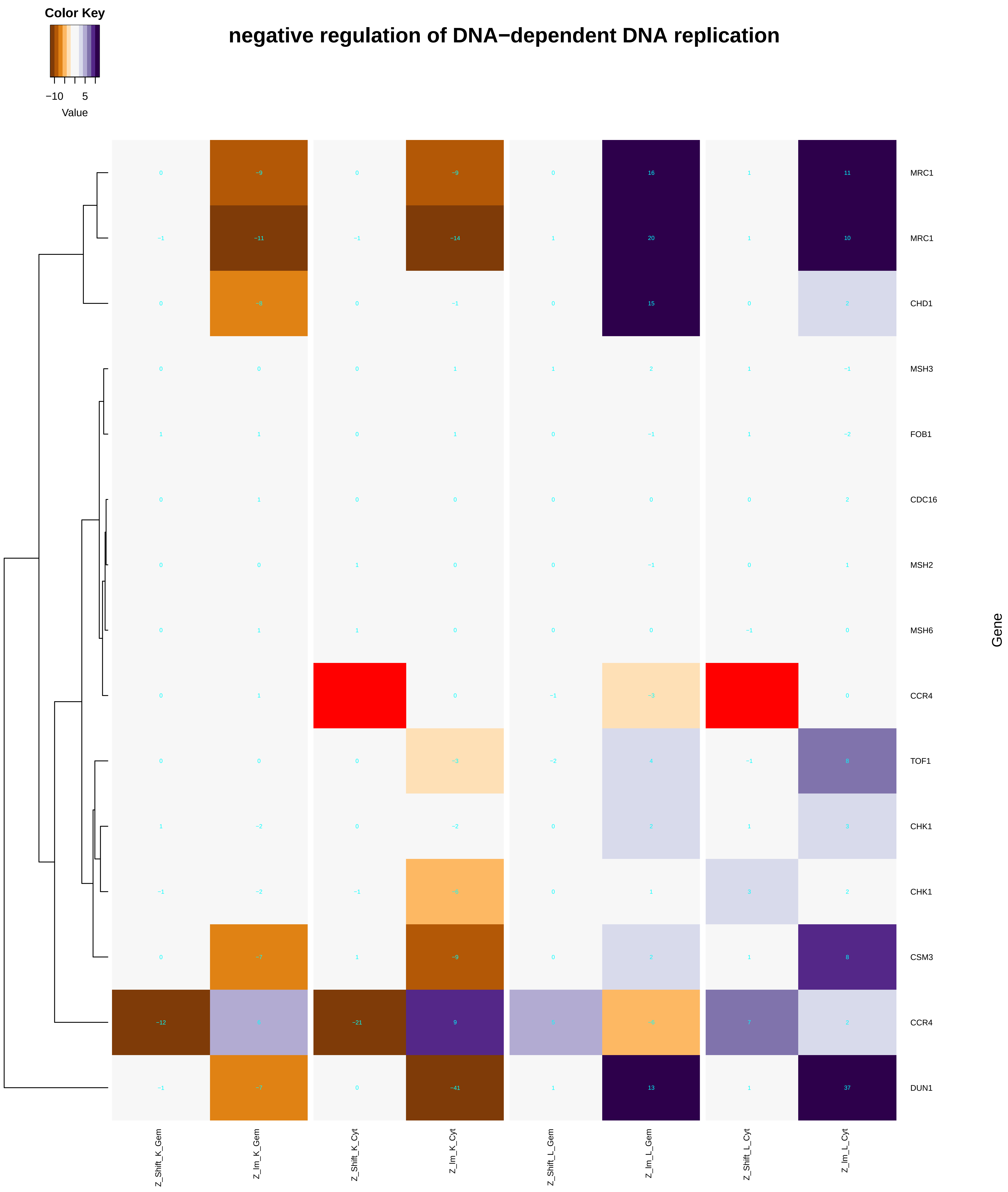
Color Key
−10
5
Value
negative regulation of DNA−dependent DNA replication
MRC1
MRC1
CHD1
MSH3
FOB1
CDC16
MSH2
MSH6
CCR4
TOF1
CHK1
CHK1
CSM3
CCR4
DUN1
Gene
Z_Shift_K_Gem
Z_Im_K_Gem
Z_Shift_K_Cyt
Z_Im_K_Cyt
Z_Shift_L_Gem
Z_Im_L_Gem
Z_Shift_L_Cyt
Z_Im_L_Cyt

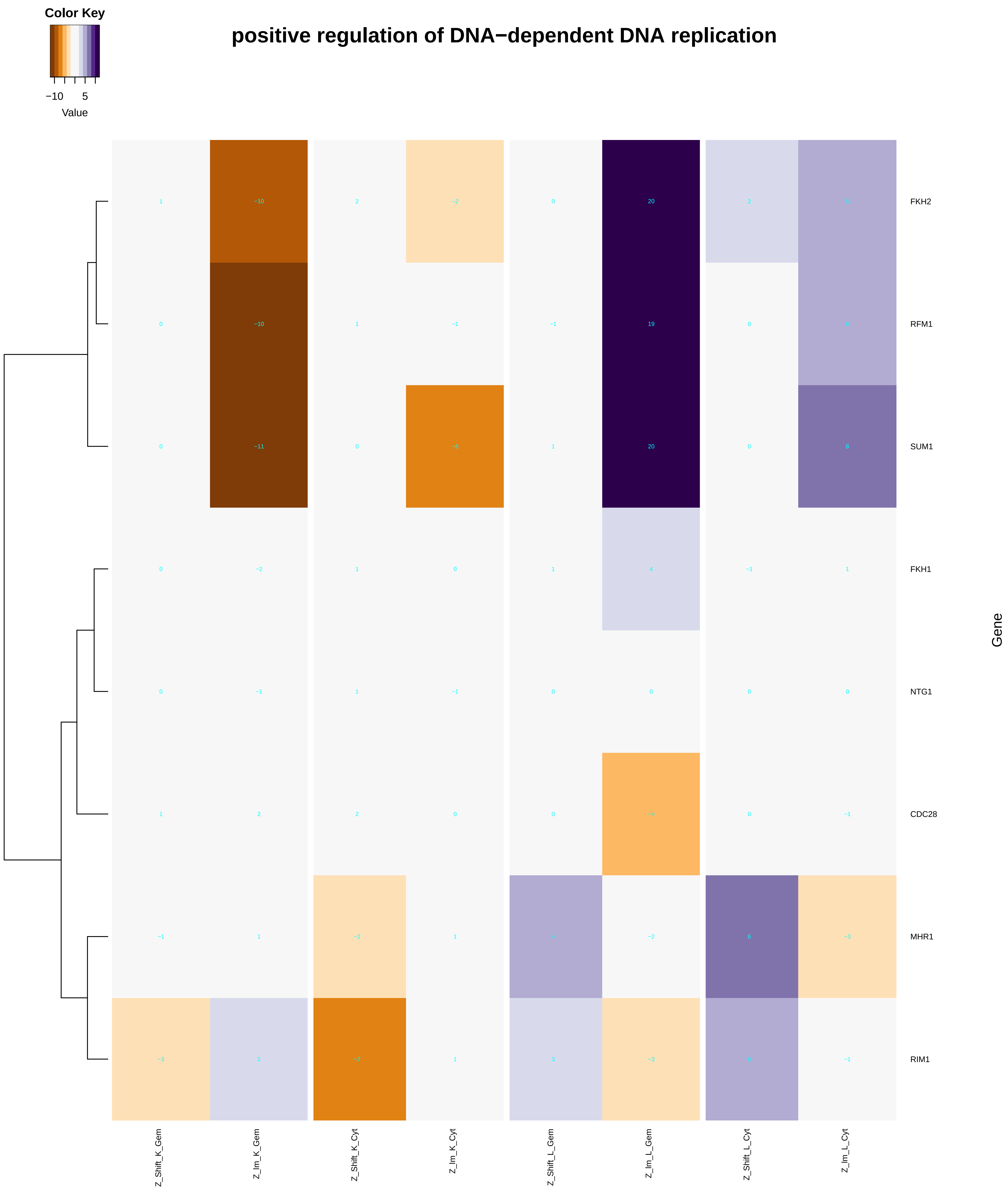

positive regulation of DNA−dependent DNA replication
Color Key
−10
5
Value
FKH2
RFM1
SUM1
FKH1
NTG1
CDC28
MHR1
RIM1
Gene
Z_Shift_K_Gem
Z_Im_K_Gem
Z_Shift_K_Cyt
Z_Im_K_Cyt
Z_Shift_L_Gem
Z_Im_L_Gem
Z_Shift_L_Cyt
Z_Im_L_Cyt

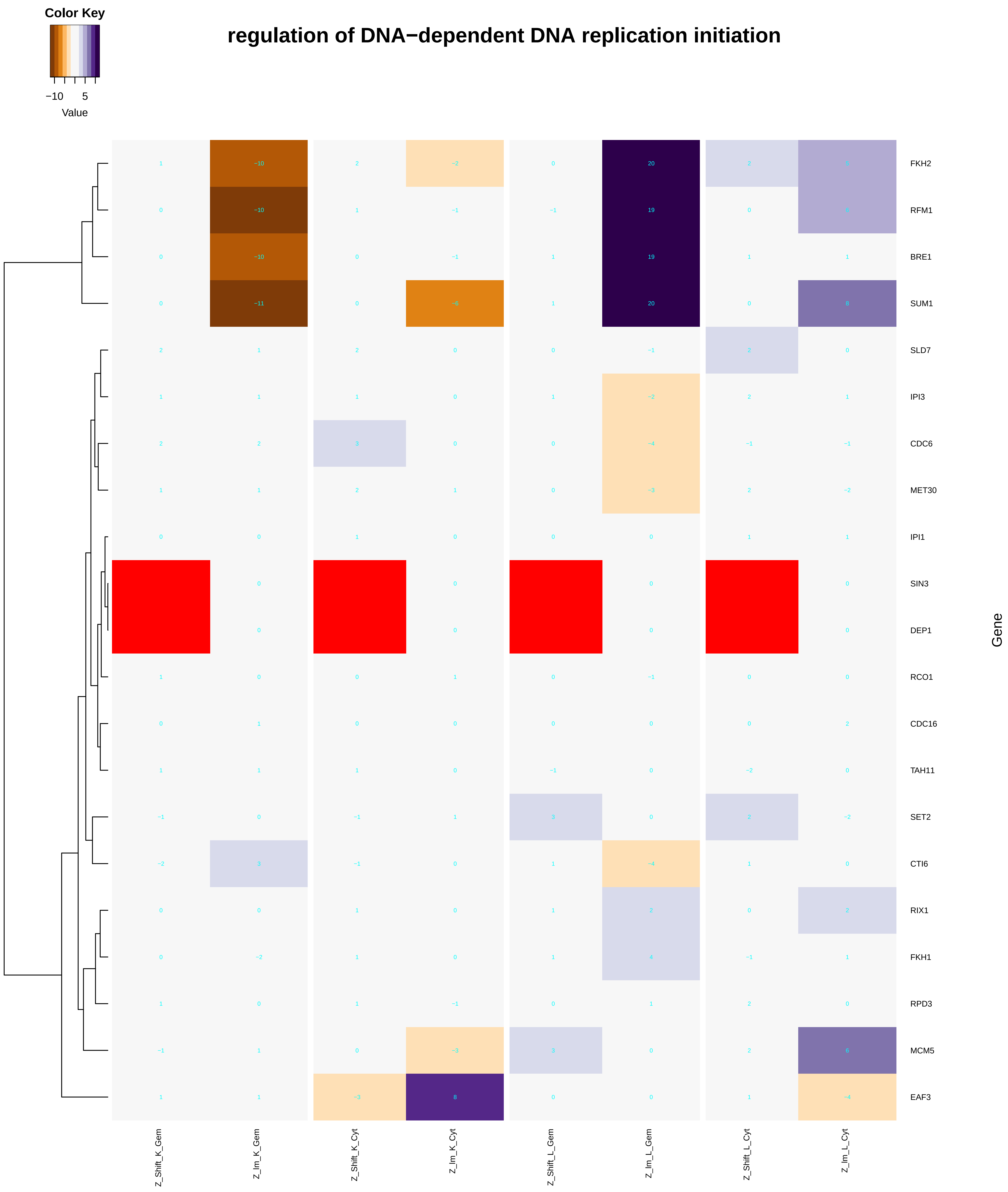
Color Key
−10
5
Value
regulation of DNA−dependent DNA replication initiation
FKH2
RFM1
BRE1
SUM1
SLD7
IPI3
CDC6
MET30
IPI1
SIN3
DEP1
RCO1
CDC16
TAH11
SET2
CTI6
RIX1
FKH1
RPD3
MCM5
EAF3
Gene
Z_Shift_K_Gem
Z_Im_K_Gem
Z_Shift_K_Cyt
Z_Im_K_Cyt
Z_Shift_L_Gem
Z_Im_L_Gem
Z_Shift_L_Cyt
Z_Im_L_Cyt

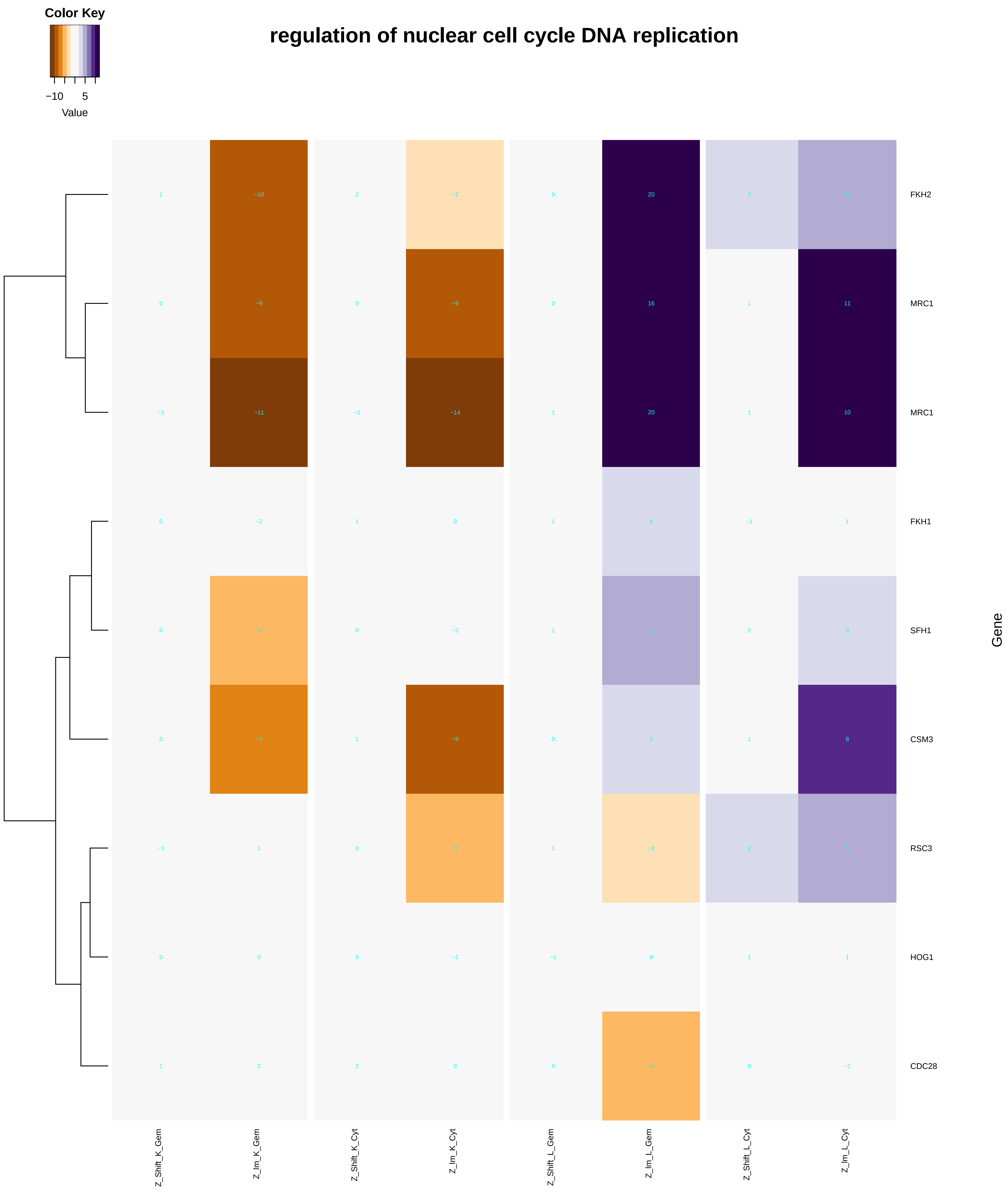
Color Key
−10
5
Value
regulation of nuclear cell cycle DNA replication
FKH2
MRC1
MRC1
FKH1
SFH1
CSM3
RSC3
HOG1
CDC28
Gene
Z_Shift_K_Gem
Z_Im_K_Gem
Z_Shift_K_Cyt
Z_Im_K_Cyt
Z_Shift_L_Gem
Z_Im_L_Gem
Z_Shift_L_Cyt
Z_Im_L_Cyt

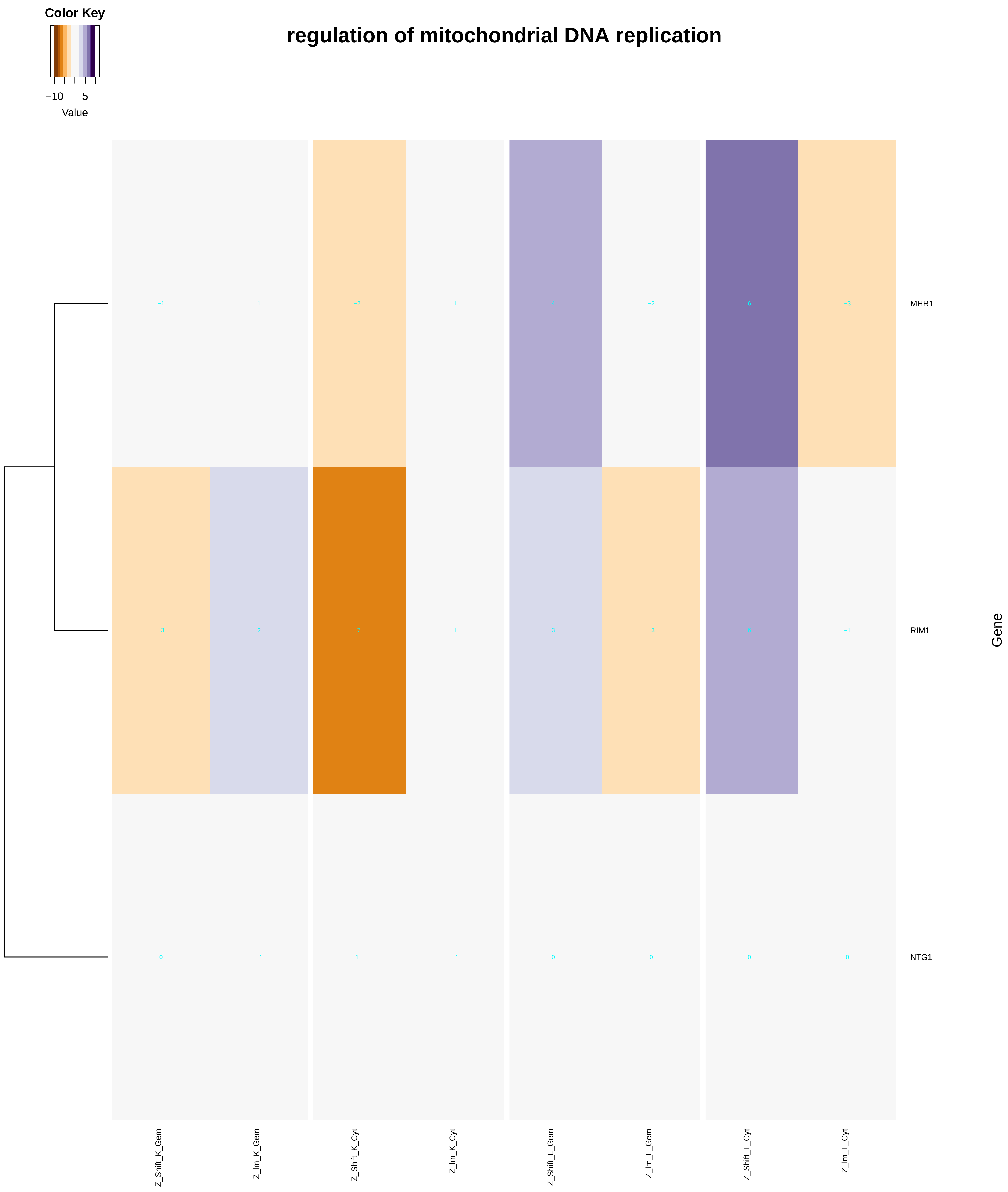
regulation of mitochondrial DNA replication
Color Key
−10
5
Value
MHR1
RIM1
NTG1
Gene
Z_Shift_K_Gem
Z_Im_K_Gem
Z_Shift_K_Cyt
Z_Im_K_Cyt
Z_Shift_L_Gem
Z_Im_L_Gem
Z_Shift_L_Cyt
Z_Im_L_Cyt

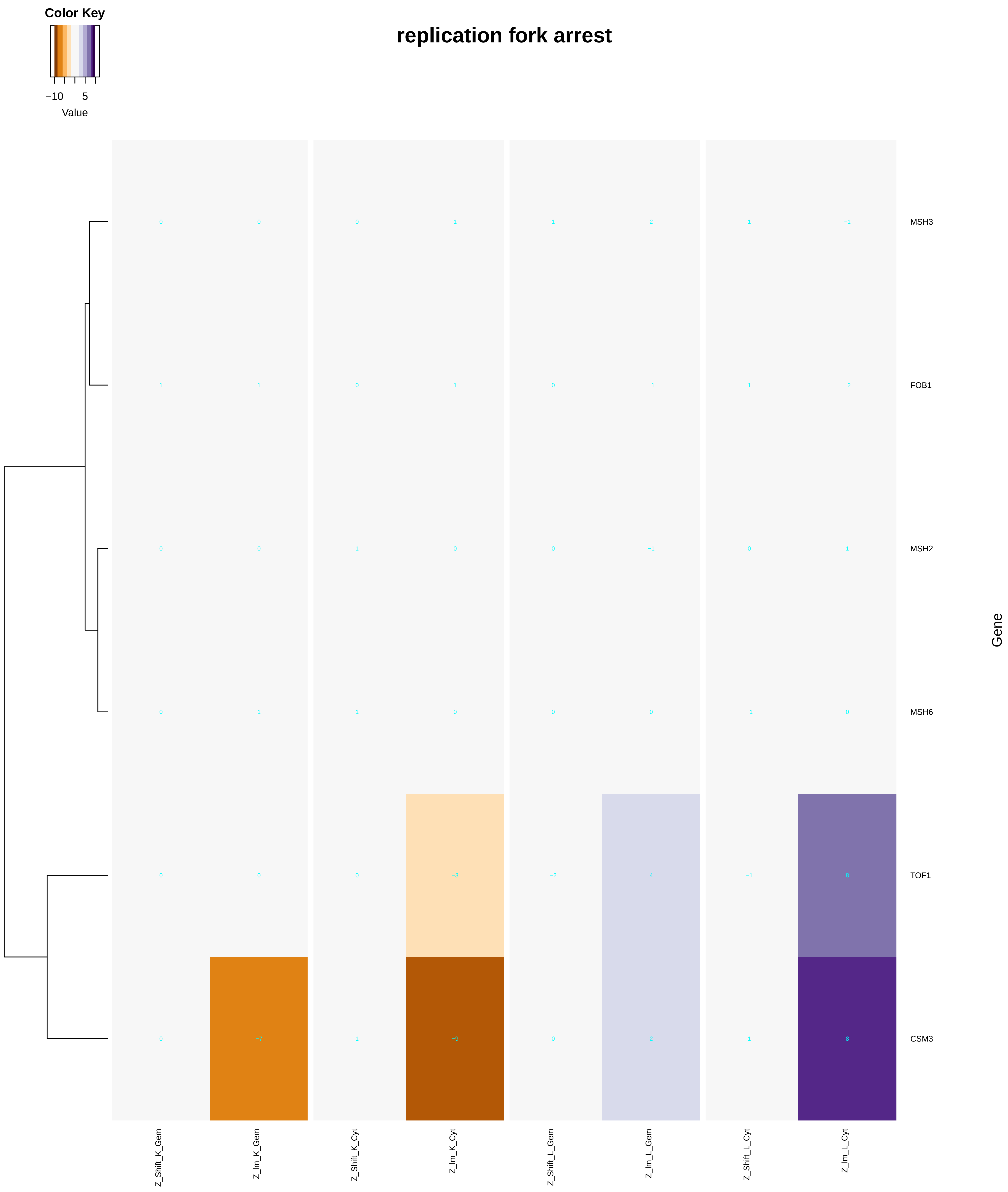
Color Key
−10
5
Value
replication fork arrest
MSH3
FOB1
MSH2
MSH6
TOF1
CSM3
Gene
Z_Shift_K_Gem
Z_Im_K_Gem
Z_Shift_K_Cyt
Z_Im_K_Cyt
Z_Shift_L_Gem
Z_Im_L_Gem
Z_Shift_L_Cyt
Z_Im_L_Cyt

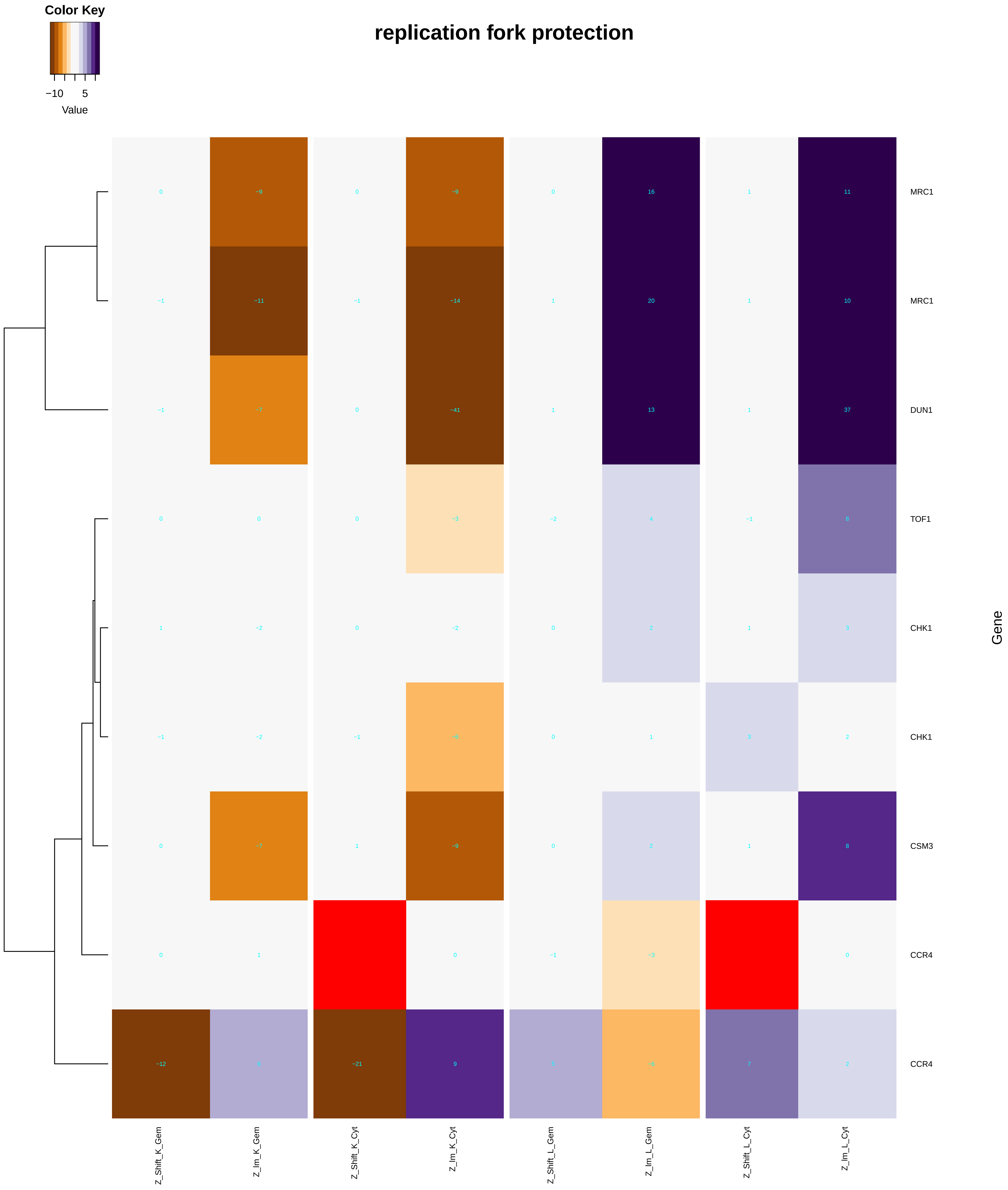
replication fork protection
Color Key
−10
5
Value
MRC1
MRC1
DUN1
TOF1
CHK1
CHK1
CSM3
CCR4
CCR4
Gene
Z_Shift_K_Gem
Z_Im_K_Gem
Z_Shift_K_Cyt
Z_Im_K_Cyt
Z_Shift_L_Gem
Z_Im_L_Gem
Z_Shift_L_Cyt
Z_Im_L_Cyt

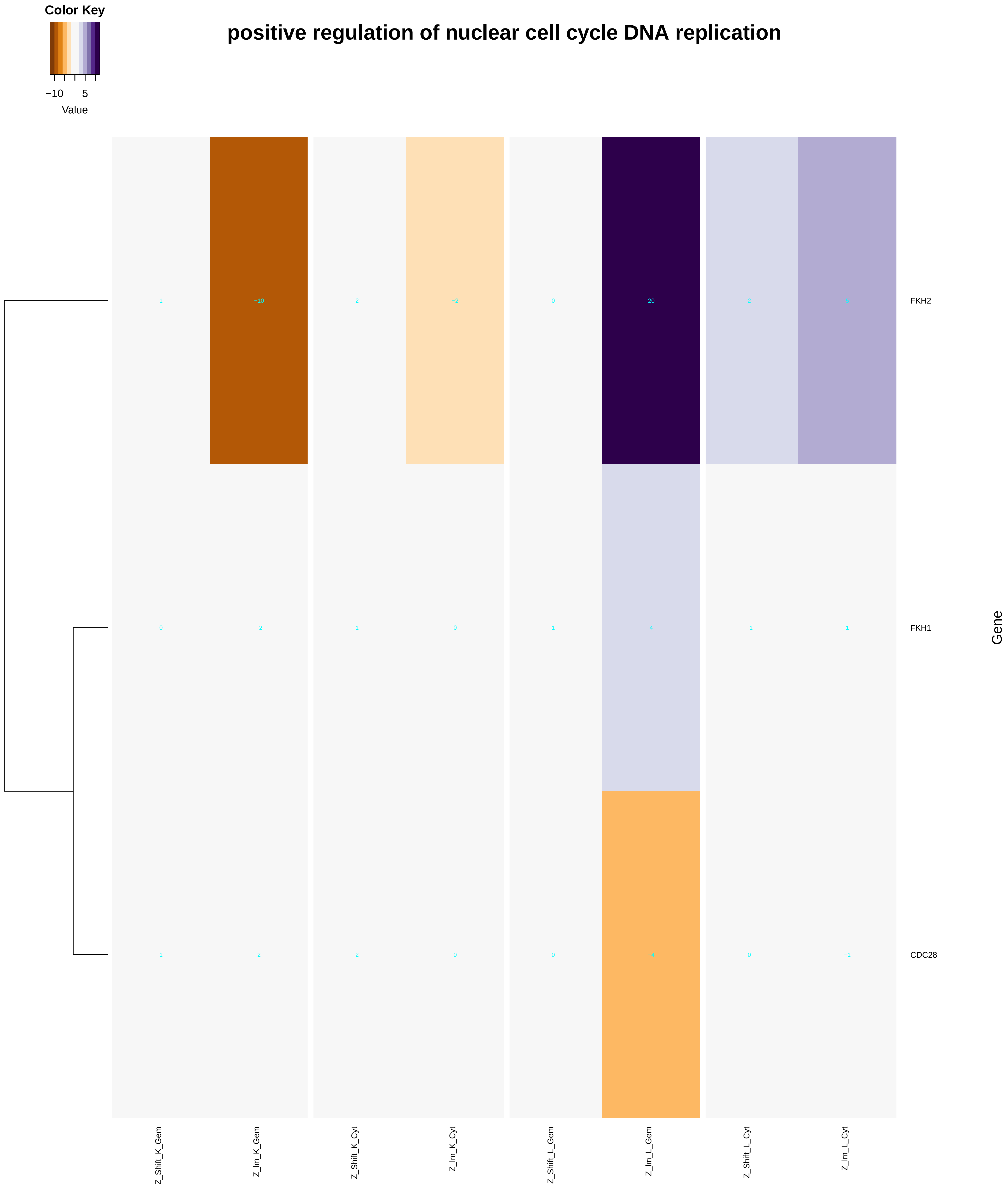
Color Key
−10
5
Value
positive regulation of nuclear cell cycle DNA replication
1
−10
2
−2
0
20
2
5
FKH2
0
−2
1
0
1
4
−1
1
FKH1
1
2
2
0
0
−4
0
−1
CDC28
Gene
Z_Shift_K_Gem
Z_Im_K_Gem
Z_Shift_K_Cyt
Z_Im_K_Cyt
Z_Shift_L_Gem
Z_Im_L_Gem
Z_Shift_L_Cyt
Z_Im_L_Cyt

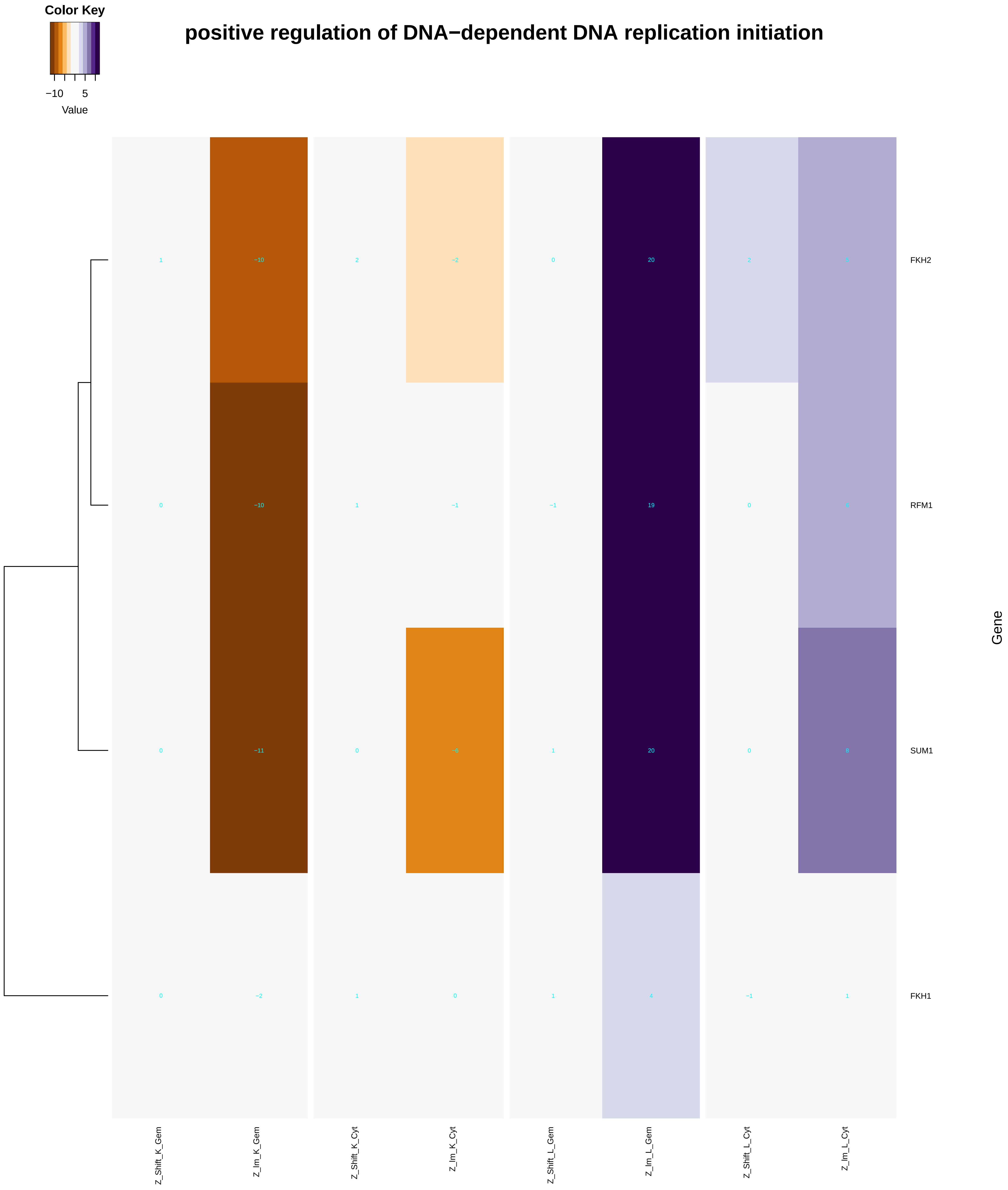
Color Key
−10
5
Value
positive regulation of DNA−dependent DNA replication initiation
1
−10
2
−2
0
20
2
5
FKH2
0
−10
1
−1
−1
19
0
6
RFM1
0
−11
0
−6
1
20
0
8
SUM1
0
−2
1
0
1
4
−1
1
FKH1
Gene
Z_Shift_K_Gem
Z_Im_K_Gem
Z_Shift_K_Cyt
Z_Im_K_Cyt
Z_Shift_L_Gem
Z_Im_L_Gem
Z_Shift_L_Cyt
Z_Im_L_Cyt

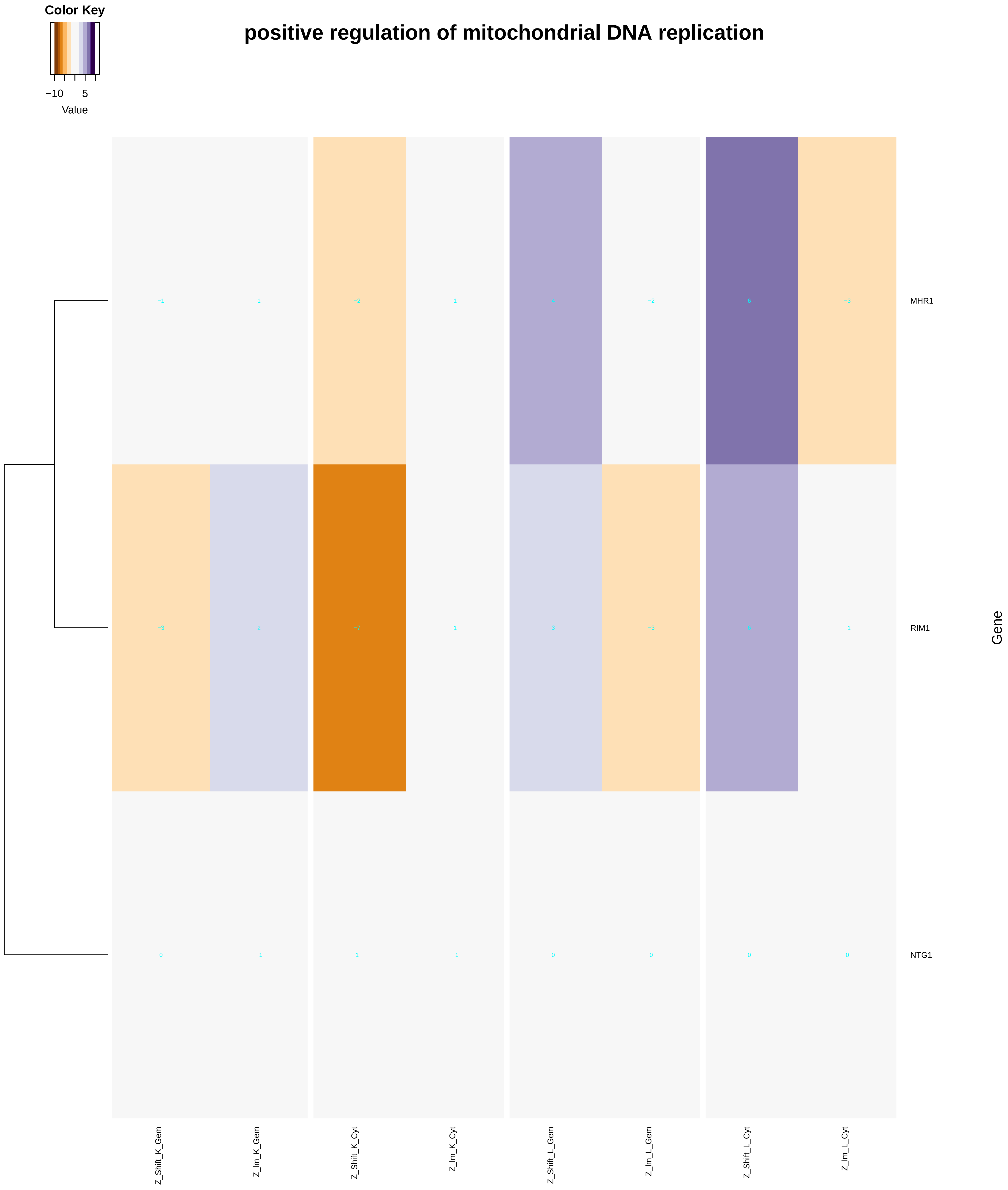

Color Key
−10
5
Value
positive regulation of mitochondrial DNA replication
MHR1
RIM1
NTG1
Gene
Z_Shift_K_Gem
Z_Im_K_Gem
Z_Shift_K_Cyt
Z_Im_K_Cyt
Z_Shift_L_Gem
Z_Im_L_Gem
Z_Shift_L_Cyt
Z_Im_L_Cyt
